# Ursolic Acid Inhibits Cigarette Smoke Extract-Induced Human Bronchial Epithelial Cell Injury and Prevents Development of Lung Cancer 

**DOI:** 10.3390/molecules17089104

**Published:** 2012-08-02

**Authors:** Wenbo Liu, Xiaobin Tan, Luan Shu, Hanyan Sun, Jie Song, Ping Jin, Siming Yu, Min Sun, Xiaobin Jia

**Affiliations:** 1 Key Laboratory of Delivery Systems of Chinese Meteria Medica, Jiangsu Provincial Academy of Chinese Medicine, Nanjing, Jiangsu 210028, China; Email: lwb870526@yahoo.cn (W.L.); njtxb@hotmail.com (X.T.); shuluan2006@hotmail.com (L.S.); momo198420@163.com (J.S.); hunanjinping@163.com (P.J.); 2 Department of Life Sciences, Anhui University, Hefei, Anhui 230039, China; Email:shyroom@126.com (H.S.); yusm08@163.com (S.Y.); sm3216@126.com (M.S.)

**Keywords:** Nrf2, CSE, phase II detoxifying enzymes, ursolic acid, chemopreventive

## Abstract

Cigarette smoking is the main cause of chronic obstructive pulmonary disease and lung cancer. The present study was aimed to explore the chemopreventive effect of ursolic acid (UA) on these diseases. In the CSE treated normal human bronchial epithelial cell model, UA alleviated cytotoxicity caused by CSE, recovered the intracellular redox balance, and relieved the stimulation of external deleterious factors as well. UA mitigated CSE-induced DNA damage through the Nrf2 (nuclear factor erythroid 2-related factor 2) pathway. Moreover, UA inhibited lung cancer development in the model established by A549 cells in nude mice *in vivo*. For the first time, our results indicate that UA could be developed as a potential lung cancer chemopreventive agent.

## 1. Introduction

Cigarettes contain many kinds of chemical constituents, and cigarette smoke includes nicotine, tar, polycyclic aromatic hydrocarbons, carbon monoxide, quinones, and other harmful substances [1,2]. These substances may be catalytically transformed by phase I metabolism enzymes (e.g., CYP450) into more toxic electrophilic compounds, which cause cellular damage and ultimately lead to pulmonary emphysema, lung cancer, and other diseases. NAPDH quinine oxidoreductase 1 (NQO1) reduces quinone to hydroquinone but avoids its conversion to hemiquinone, which has adverse effects. Glutathione transferase (GST) can be catalyzed by reduced glutathione (GSH) and combinations of electron affinity substances and are then excreted. These two phase II detoxification enzymes may render electrophilic substances catalytic and reduce their toxicity, thereby preventing tumorigenesis [3,4,5,6,7]. Recent research has shown that the Nrf2 (nuclear factor erythroid 2-related factor 2) signaling pathway contains crucial phase II detoxification enzymes in its upstream channel [8,9]. Induction of Nrf2 signaling by pharmacologic agents or endogenous cellular stress, for example, conjugated polyenes, heavy metals and chemoprevention agent.

Lung cancer, including small cell lung cancer and non-small cell lung cancer, is the major cause of cancer-related mortality worldwide. Non-small cell lung cancer, which includes large cell carcinoma, adenocarcinoma, squamous cell carcinoma, and some other subtypes, accounts for some 80% of lung cancer cases. It can be caused by tobacco smoke, viral infection, and ionizing radiation. In spite of major breakthroughs in diagnostic tests, surgical techniques, and the development of new chemotherapy drugs, the overall 5-year survival rate of non-small cell lung cancer patients remains at approximately 10%–15%. Therefore, the importance of early prevention and treatment needs to be emphasized.

Ursolic acid (UA; also known as tripterine) is widely distributed in loquat leaves, glossy privet leaves, forsythia, *Prunella vulgaris*, and other traditional Chinese medicines. It shows hepatoprotective, antioxidant, and antitumor effects and exhibits low toxicity [10,11,12]. Previous studies have shown that UA has inhibitory actions against nasopharyngeal carcinoma, gastric cancer, esophageal cancer, and liver cancer [13,14,15,16,17]. This study investigated the cytoprotective effect of UA in normal human bronchial epithelial (NHBE) cells treated with cigarette smoke extract (CSE). It was observed that UA reduced CSE-induced DNA damage and intracellular stress response. Our finding suggested UA would be a promising chemopreventive agent for lung cancer therapy.

## 2. Results and Discussion

### 2.1. UA Reduces CSE-Induced Cytotoxicity in NHBE Cells

**Figure 1:**
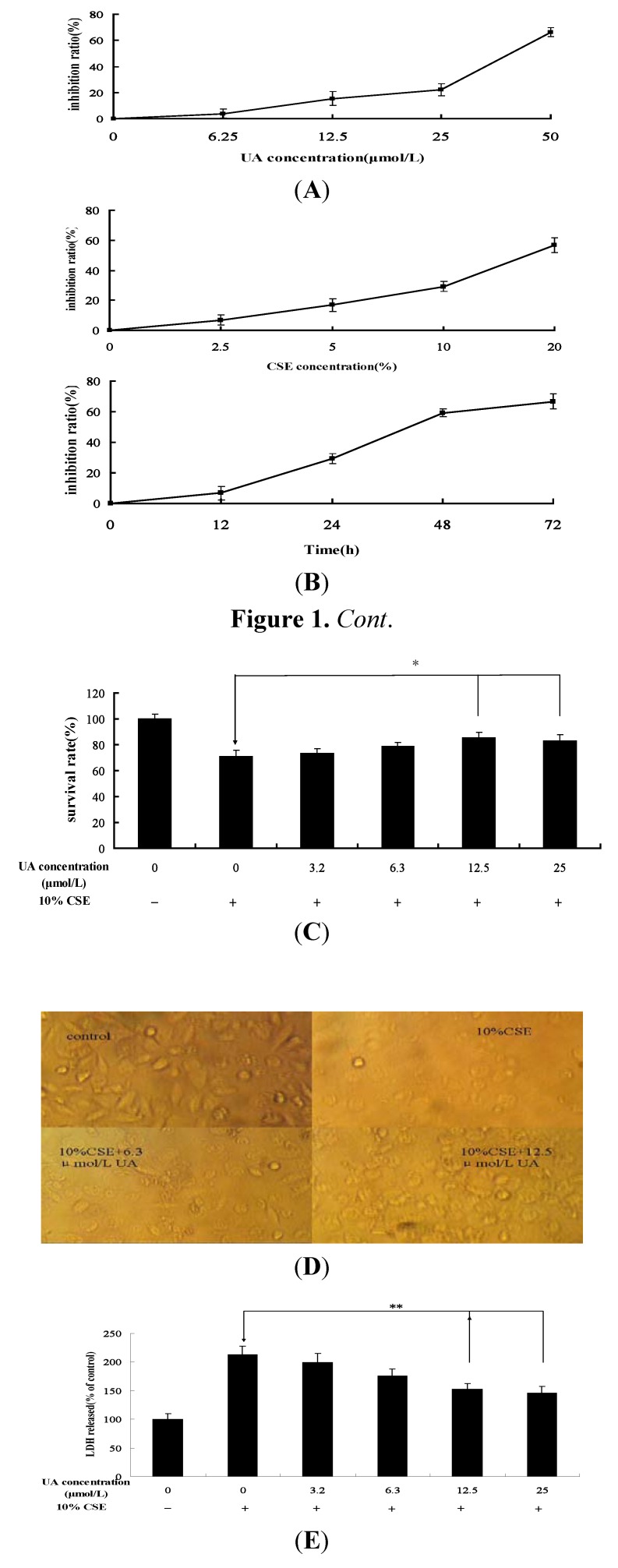
Cytoprotective effect of UA against CSE. (**A**) Effects of different concentrations of UA on NHBE cell proliferation; (**B**) Effects of different concentrations and treated time of CSE on NHBE cell proliferation; (**C**–**E**) Protective effect of UA determined through (**C**) MTT assay; (**D**) morphological examination; and (**E**) lactate dehydrogenase release. Values are expressed as mean ± SEM (n = 5). * *p* < 0.05, ** *p* < 0.01 *vs*. CSE group.

We used the 3-(4,5-dimethylthiazol-2-yl)-2,5-diphenyltetrazolium bromide (MTT) assay to investigate the effect of UA on NHBE cells. The results showed that UA can cause the inhibition of proliferation of NHBEsin high concentration (Figure 1A). Then we studied the effect of CSE on NHBE cell death. We exposed NHBE cells to CSE (2.5% to 20%) for 24 h and 10% CSE for 12 h, 24 h, 48 h, 72 h measured the cytotoxicity of CSE by MTT assay. As shown in Figure 1B, CSE reduced the viability of NHBE cells in a dose-dependent and time-dependent manner; the inhibition rates of NHBE cells treated with 2.5%, 5%, 10%, and 20% CSE solutions were 6.77 ± 3.32%, 16.86 ± 4.18%, 29.27 ± 3.29%, and 56.73 ± 4.87%, respectively. We next investigated the protective effect of UA on CSE-induced cell death and simultaneously administered various concentrations of UA and 10% CSE to NHBE cells. The MTT assay showed that UA significantly reduced the CSE-induced toxicity of NHBE cells (Figure 1C) and the morphological cytology of the treatment groups (Figure 1D). The cell survival rates of the groups treated with 3.2, 6.3, 12.5, and 25 µmol/L UA were 73.29 ± 3.84%, 78.67 ± 2.96%, 85.43 ± 4.06%, and 86.22 ± 4.47%, respectively. UA also significantly reduced the CSE-induced lactate dehydrogenase excessive efflux in a dose-dependent manner compared with untreated cells (Figure 1E). Differences between the CSE group and the groups treated with 12.5 and 25 µmol/L UA were statistically significant (*p* < 0.05).

The results of this part demonstrated that CSE cytotoxicity and oxidative effects, enhanced cell injury and depress the cell viability in NHBEs. In addition, the results showed that UA alleviates CSE-induced cytotoxicity.

### 2.2. UA Palliates CSE-Induced GSH Content Reduction

**Figure 2:**
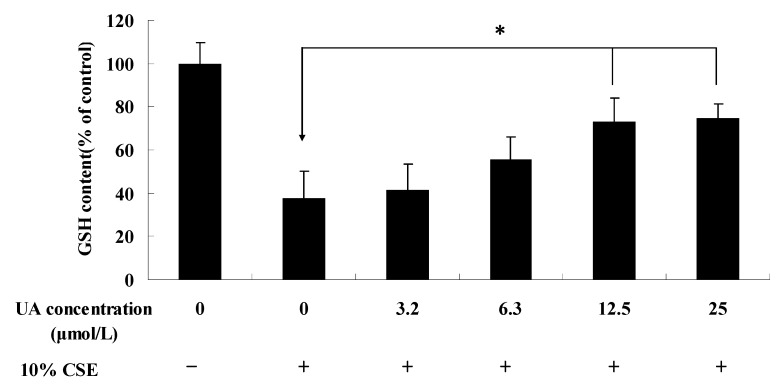
Effect of UA on CSE-induced total cellular GSH content. Data are expressed as mean ± SD (n = 5). * *p* < 0.05 *vs*. CSE group.

The GSH level of NHBE cells exposed to 10% CSE 24 h was remarkably decreased (37.83 ± 12.56% of control) in parallel with the control cells, and UA alleviated the CSE-induced GSH reduction in a dose-effect manner (Figure 2). With the addition of CSE, the intracellular GSH content decreased, the UA concentration increased, and the GSH content gradually recovered. the content of GSH in NHBE cells reduced to 41.28 ± 12.33%, 55.39 ± 10.68%, 72.84 ± 11.32%, 74.96 ± 6.19% of control, while the concentration of UA increased to 3.2, 6.3, 12.5, and 25 µmol/L, respectively. Differences between the CSE group and the groups treated with 12.5 and 25 µmol/L UA were statistically significant (*p* < 0.05).

The findings also echo previous research on the effects in pulmonary cells. In our study, we used CSE-treated NHBEs experimental model to research the cytoprotective effects of UA. We found that, while UA treatment remarkably palliates CSE-induced increases in intracellular damage, it also alleviated the CSE-induced promotion of enzyme activity and GSH content in NHBEs.

### 2.3. UA Alleviates CSE-Induced GST and NQO1 Activity and Protein Expression

Nrf2 is a key regulator of the inducible expression of phase II detoxifying enzymes that includes GST and NQO1. Exogenous agents and endogenous cellular stress such as sulforaphane, epigallocatechin gallate (EGCG) and conjugated polyenes induce the Nrf2 signal pathway. We further studied the effect of CSE and PVE on Nrf2 and phase II detoxifying enzymes protein expression in NHBEs.

**Figure 3:**
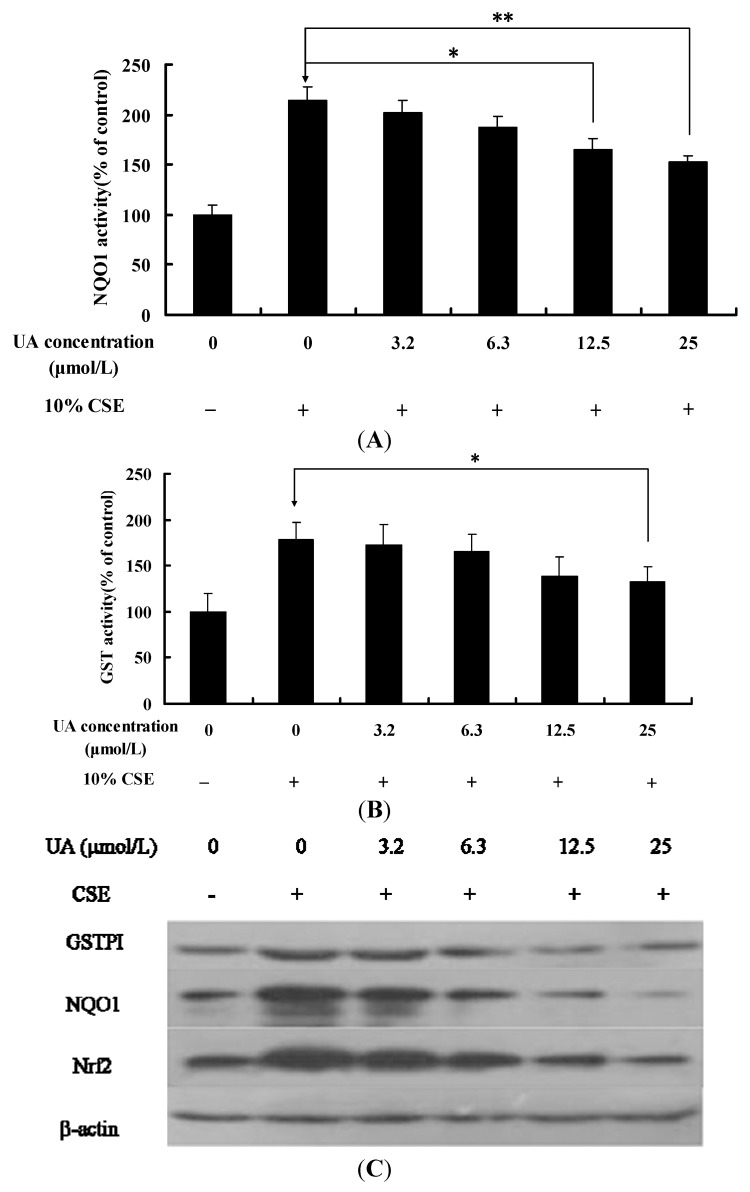
Effect of UA on the activity of phase II enzymes (**A**) NQO1 and (**B**) GST and (**C**) the expression of Nrf2, NQO1, and GSTPI in NHBE cells. * *p* < 0.05, ** *p* < 0.01 *vs*. CSE group.

Cells were treated with various concentrations of CSE for 24 h to determine the effects of CSE treatment on the expression of GST and NQO1. The enzyme activities of GST, NQO1, and GSTPI and the protein expression of NQO1 and Nrf2 were determined by GST and NQO1 activity assay and Western blot analysis, respectively. As shown in Figure 3A,B, CSE induced protein overexpression and activity of GST and NQO1.

Moreover, UA could not palliate the CSE-induced protein expression and enzyme activity at 3.2 and 6.3 µmol/L, respectively, but it significantly alleviated these at the corresponding concentrations of 12.5 and 25 µmol/L (Figure 3C). In addition, UA significantly reduced CSE-induced Nrf2 protein overexpression.

### 2.4. UA Palliates CSE-Induced DNA Damage

**Figure 4:**
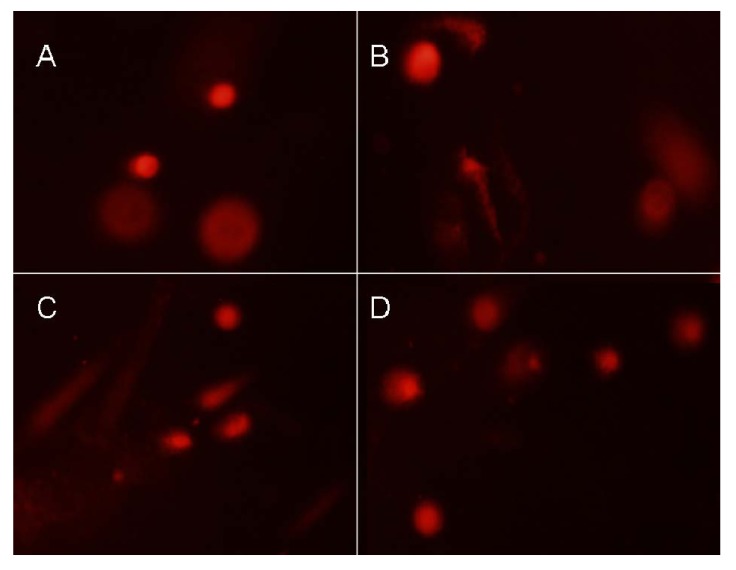
Representative photomicrographs showing the DNA migration pattern in NHBE cells: (**A**) control group; (**B**) treatment group with 10% CSE for 24 h; (**C**) treatment group with 10% CSE + UA (6.3 µmol/L) for 24 h; (**D**) treatment group with 10% CSE + UA (12.5 µmol/L) for 24 h.

Recently it was reported that low concentrations CSE only induced DNA damage in human bronchial epithelial cells and not cell apoptosis [18,19]. We examined the effect of UA on CSE-induced DNA damage in NHBE cells. Comet assay was performed to analyze the CSE-induced DNA damage after NHBE cells were incubated with 10% CSE for 24 h. Treatment with CSE led to a significant increase in DNA damage. The tail length treated with CSE was significantly longer compared with control, and UA treatment led to a significant reduction in the DNA damage. As shown in Figure 4, UA significantly reduced CSE-induced DNA damage (*p* < 0.05).

### 2.5. UA Inhibits Tumorigenesis *in Vivo*

**Figure 5:**
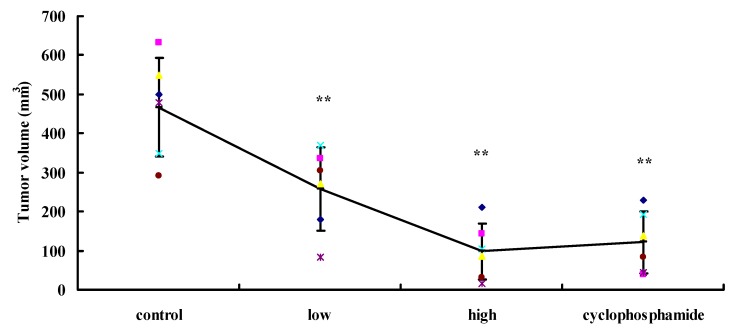
Effect of UA on tumorigenesis of lung tumor cells *in vivo*: normal control group, experimental group treated with 2.5 mg/kg/day and10 mg/kg/day UA and cyclophosphamide positive control group (n = 6). ** *p* < 0.01 *vs*. Control group.

We determined the inhibitory effect of UA in a nude mouse transplantable model of lung cancer cell. After treatment, the tumor burden was statistically lower in UA-treated mice compared with the vehicle-treated group. Cyclophosphamide was used as a positive drug. As shown Figure 5, the tumor volumes in the UA groups were smaller than those in the control group.

## 3. Experimental

### 3.1. Chemicals

UA (purity, >98%) was purchased from the National Institute for the Control of Pharmaceutical and Biological Products (Beijing, China). RPMI-1640 medium and COMET DNA Damage Detection Kit were obtained from Nanjing KeyGen Biotech. Co. Ltd. (Nanjing, China). PMSF, MTT, and dimethyl sulfoxide were purchased from Sigma-Aldrich (St. Louis, MO, USA).

### 3.2. Cell Culture

NHBE cells were obtained from American Type Culture Collection (Rockville, MD, USA). They were maintained in RPMI-1640 medium supplemented with 10% fetal bovine serum (Gibco, Carlsbad, CA, USA), 100 U/mL of penicillin, and 100 U/mL of streptomycin in a humidified incubator at 5% CO_2_ and 37 °C.

### 3.3. Preparation of CSE

Commercial cigarettes were smoked using a vacuum pump for 5 min each, and smoke per 10 mL of serum-free cell growth medium was used to generate 100% CSE-PBS solution. The CSE was prepared 30 min before use, and the pH was regulated to 7.4. The solution was sterilized by filtration with 0.22 µm of filter membrane [20,21,22,23].

### 3.4. Cytotoxicity and Viability Assays

Cells were inoculated in 96-well plates at a density of 5 × 10^4^ cells/mL for 24 h and then treated with UA (6.3, 12.5, 25, and 50 µmol/L) or/and CSE (2.5%–20%) but without fetal bovine serum for 12 h, 24 h, 48 h and 72 h. Afterwards, MTT was added to the medium for an additional 4 h. After the supernatant was discarded, 100 mL of dimethyl sulfoxide was added to the mixture to dissolve the formazan crystals with 10 min vibration. Optical density was measured by a microplate reader at 570 and 630 nm. The growth inhibitory ratio of the treated cells was calculated as follows:



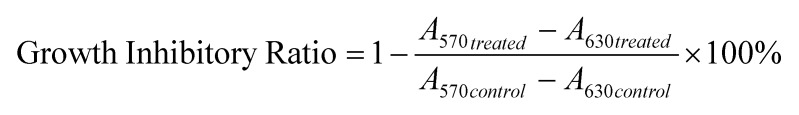


### 3.5. Estimation of DNA Damage by Comet Assay

The protective effect of PVE on CSE-induced NHBE cell DNA damage was investigated by comet assay. Cells were washed with ice-cold PBS and harvested. Comets were determined using a COMET DNA Damage Detection Kit and visualized with a fluorescence microscope. Briefly, cells were incubated with or without CSE or PVE for 24 h. Subsequently, the cells were harvested and then fixed with 4% paraformaldehyde for 1 h.

### 3.6. Lactate Dehydrogenase Content

Lactate dehydrogenase release was detected using a biochemical analyzer (Roche Modular, Basel, Switzerland). Two hundred microliters of culture medium was taken out and measured immediately.

### 3.7. Whole Cell Lysate Preparation

Treated and untreated cells were washed two times with 4 °C PBS and lysed in cell lysis buffer (Biyuntian Biotech. Co. Ltd., Nantong, China) with PMSF at 4 °C for 30 min. The whole cell lysates were centrifuged at 13,000 rpm for 5 min at 4 °C, and the supernatant solution was stored at −20 °C for the subsequent experiments. The concentration of extracted proteins was determined using a Nanodrop 1000 Spectrophotometer (Thermo, San Jose, CA, USA).

### 3.8. Measurement of Intracellular GSH Levels

Intracellular GSH was measured in cell lysates using a GSH detection kit (Nanjing KeyGen Biotech. Co. Ltd., Nanjing, China) in accordance with the manufacturer’s protocol.

### 3.9. Analysis of Detoxification Enzyme Activity

NQO1 activity was measured as described previously [24]. The reaction buffer was composed of 50 mM Tris-HCl (pH 7.5), 0.25 mM NADPH (Roche, Basel, Switzerland), and 80 µM 2,6-dichlorophenolindophenol (TCI, Tokyo, Japan). Five microliters of cell lysates was added to 0.9 mL buffer, and enzyme activity was measured by spectrophotometry at a wavelength of 600 nm for 3 min. The reduction of absorbance per minute per milligram of protein indicated the vitality of NQO1. The GST activity of the cell lysates was then measured using a GST vitality detection kit (Nanjing KeyGen Biotech. Co. Ltd., Nanjing, China) following the manufacturer’s instructions.

### 3.10. Western Blot Analysis

Cell lysates (50 µg protein) were subjected to 15% SDS-polyacrylamide gels and transferred onto a PVDF (polyvinylidene fluoride) membrane. The membranes were blocked with TBST + 5% bovine serum albumin for 2 h at room temperature. After washing with TBST buffer, the membranes were incubated with first antibody (Santa Cruz Biotechnology, Santa Cruz, CA, USA) overnight at 4 °C. The membranes were then incubated with the secondary antibodies for 1 h after being washed three times with TBST at room temperature on a shaking table. Enhanced chemiluminescence (ECL) was used for protein detection.

### 3.11. Effect of UA on Tumor Growth in Nude Mice

6 to 8-week-old nude mice were obtained from Shanghai Laboratory Animal Commission (Shanghai, China). A total of 10 mg/kg of UA was intragastrically administered to the UA group for 1 week. A549 lung cancer cells were cultured and resuspended in PBS and then injected subcutaneously into the right flank of the nude mice with cell suspension (1 × 10^6^) in the second week. Four weeks after injection, the tumors were excised and measured.

### 3.12. Statistical Analysis

Statistical analysis was performed using SPSS 16.0 software, and the data were expressed as mean ± SD. Comparisons between groups were analyzed using *t* test. *p* < 0.05 was considered significant, whereas *p* < 0.01 was considered highly significant.

## 4. Conclusions

Our present study found that CSE induced an increase in the activity of cellular phase II detoxification enzymes as well as the protein expression levels, while it reduced GSH content and DNA damage. It was observed for the first time that UA protected against CSE-induced cell injury and suppressed lung tumor growth *in vivo*. Taken together, these results indicated that UA could play an important role in the prevention and treatment of lung tumors. We conclude that UA may be beneficial as a food additive with antitumor or tumor-preventive effects.
